# History of a Case of Excision of a Schirrous Tumor from the Uterus

**Published:** 1820-06

**Authors:** Giuseppe Giorgi

**Affiliations:** Chirurgo primario condotto dell'illustre Città d'Imola, Maestro di Ostetricia, e Socio di molte Accademie Scientifiche e Letterarie.


FOR THE LONDON MEDICAL AND PHYSICAL JOURNAL.

### *History of a Case of Excision of a Schirrous Tumor from the Uterus.*

By Sig. Giuseppe Giorgi, Chirurgo primario condotto dell'illustre Città d'Imola, Maestro di Ostetricia, e Socio di molte Accademie Scientifiche e Letterarie.

M. G. COLPISASI, forty-four years of age, of a slender habit of body and a sensible and melancholic temperament, the mother of several children, and who had led a sedentary and unhappy life, suffered for many years, at the time of menstruation, pains in the internal parts of the genital organs, which continued as long as the menses flowed. The malady continued in this way until the fortieth year of her age; when menorrhagia came on, accompanied with all the symptoms of asthenia. She now began to feel a weight and sense of pruritus in the vagina, with sudden darting pains, which often awaked her when they occurred during sleep: pains of this kind would come on and subside several times during the night. She was at this time under the care of my colleague, Dr. Cherubini, of Orvieto, who had employed all the usual means for the relief of such cases, without benefit. She was visited by several other professors, who considered that she had a cancerous affection of the uterus, and declared her disease to be incurable. She was in this state, when a large fleshy body presented at the orifice of the vagina, and prevented the evacuation of the urine. I was called to visit the patient on the 3d of May, 1814, by her physician; when I found her in bed, in a state of extreme debility. On examining the affected parts, I found a fleshy body, bedewed with blood, of the size of the head of a fetus; not perfectly round, having several protuberances on its surface; of a stony hardness; and which presented without the vagina to the distance of nearly the breadth of three fingers.

From the antecedent symptoms, described by the patient, as well as from the account of the physician who had attended her, I concluded that it was probably a schirrus, having its seat either in the neck or mouth of the uterus; and that the hemorrhage which the patient had suffered came from this part, the vessels about it being in a state of dilatation, and opening on its surface. On further examination, I perceived my conjecture was well founded, and that the rest of the schirrus occupied the vaginal canal. The circumstances of the case being urgent, and decisive conduct, founded on practical experience, being necessary, I proposed to remove the parts by extirpation; knowing that such an operation had been several times performed with favourable results. This was immediately consented to.

The patient was placed on one side of the bed, as in the act of parturition, with the nates a little raised, the thighs parted, and she was supported in this way by the attendants. Being satisfied, by another examination, that the schirrus occupied the mouth of the uterus, I introduced between the schirrus and the vagina the blades of Smellie's forceps, and drew down by their aid the rest of the schirrous tumor, so as to bring the orifice of the uterus quite without the orifice of the vagina; when the instruments were removed, and the parts retained in their present situation by the hands of an assistant. I then passed the index and middle finger of the left band into the vagina, and with the right hand introduced a probe-pointed bistoury along these, and carefully excised the whole of the schirrous parts from the rest of the uterus. Considerable hemorrhage took place, which was treated by the introduction of pledgets of lint dipped in a styptic liquor, and rolled in powder of agaric and dragon's-blood, with which the vagina was filled. A piece of strong thread was tied to each pledget, in order that it might not be retained in the vagina longer than was necessary. Compresses and a T bandage were applied externally, and thus the hemorrhage was suppressed. The patient was then placed in bed, in the horizontal posture, with pillows under the nates, and the thighs elevated. Repose and quiet were ordered, and a spoonful of tincture of canella directed to be administered internally. The following were the symptoms which came on after the operation:

The patient complained of an intolerable smarting sensation, and convulsive twitchings came on, which continued only for three-quarters of an hour; but the smarting lasted in a less severe degree throughout the rest of the day. No other morbid symptoms appeared. On visiting her on the following morning, I found that nothing unfavourable had happened during the night: no hemorrhage had occurred; and the patient only complained of pain and the smarting sensation. Nothing remarkable took place on the third and fourth days: the smarting had diminished; the patient only complained of the severe restriction in her diet to which she had submitted. Towards the afternoon of the fifth day, a slight fever manifested itself, when it was found that suppuration had commenced. The night, however, was passed very well, and in the course of this time the fever disappeared. On the following morning the dressings were removed for the first time, when some fetid purulent matter issued from the vagina; but no hemorrhage occurred. A decoction of china-root was injected, and the pledgets removed, which were now moistened with the same decoction and some innocent balsam. No other unfavourable symptoms occurred: the pain and smarting had disappeared. The china-root in substance was administered internally, in doses of a drachm, in a little wine, every two hours; and this medicine was continued until the tenth day. The seventh day was passed in a favourable manner. A slight paroxysm of fever recurred on the eighth day, accompanied with slight convulsive movements of a few moments' duration; but neither of these affections were present on the tenth. The quantity of food was now increased. The medicine above mentioned was continued morning and evening until the fifteenth. The patient gradually recovered her strength, which was favoured by the use of a moderately nutritious diet and more spirituous drink. The injections of decoction of china-root, and the insertion of pledgets moistened with aqua phagedenica, were continued until the completion of the cure, which was effected on the thirtieth day, when the parts were perfectly cicatrized. It is worthy of remark, that, for three months after the operation, the menses recurred in the same manner as if the uterus had not been subjected to any operation.

The woman now enjoys perfect health, and has recovered from the constitutional symptoms that had been produced by the presence of the tumor.

